# British Thyroid Association Survey of Graves' Disease Management in the UK

**DOI:** 10.1111/cen.15266

**Published:** 2025-05-08

**Authors:** Michael Atkinson, Medha Agrawal, Koteshwara Muralidhara, Prakash Abraham, Bijay Vaidya, Onyebuchi E. Okosieme

**Affiliations:** ^1^ Swansea Bay University Health Board Swansea UK; ^2^ Aneurin Bevan University Health Board Newport UK; ^3^ Kingston Hospital NHS Foundation Trust London UK; ^4^ Endocrinology, Aberdeen Royal Infirmary Aberdeen UK; ^5^ Royal Devon & Exeter Hospital University of Exeter Medical School Exeter UK; ^6^ Cwm Taf Morgannwg Health Board Merthyr Tydfil UK; ^7^ Thyroid Research Group, Systems Immunity Research Institute Cardiff University School of Medicine Cardiff UK

**Keywords:** antithyroid drugs, Graves' disease, hyperthyroidism, practice survey, radioactive iodine

## Abstract

**Background:**

Recent years have seen changes and uncertainties in evidence and guideline recommendations in Graves' disease treatment. To understand the impact of these developments on current practice, we undertook a survey of Graves' disease management in the United Kingdom and compared this to other national and international surveys.

**Method:**

Members of the British Thyroid Association, the UK Society for Endocrinology and regional endocrinology networks, were invited by e‐mail to complete a 15‐min online survey (October 2022 to March 2023).

**Results:**

Out of 158 eligible respondents, 99% were endocrinologists. For a 40‐year‐old female with a first presentation of Graves' hyperthyroidism, TSH‐receptor antibodies (TRAb) were requested at diagnosis and at follow‐up by 95% and 76%, respectively. Isotope scans and ultrasound were rarely requested (< 5%). Majority (95%) would treat with antithyroid drugs (ATD), predominantly Carbimazole (CMZ), while radioactive iodine (RAI) was preferred for recurrent disease (81%). Common reasons for avoiding RAI were thyroid eye disease, pregnancy intention, or contact with young children whereas biochemical severity, goitre, or male sex did not influence decision to use RAI. Propylthiouracil (PTU) was preferred in preconception and early pregnancy, but after the first‐trimester, 50% would continue PTU while 50% switch back to CMZ.

**Conclusions:**

The survey confirms a growing application of TRAbs, both for diagnostic and prognostic purposes. ATDs remain the preferred first‐line therapy for Graves' disease, which is consistent with global trends but contrary to National Institute of Health and Care Excellence (NICE) guidance. Further studies are required to explore the clinical and pragmatic determinants of current treatment approaches.

## 1 Introduction

Graves' disease affects about 0.5%–1.0% of the population, and is predominantly more common in women [1, 2]. The pathological hallmark is the presence of circulating thyroid stimulating hormone receptor antibodies (TRAbs) which induce hyperthyroidism through stimulation of its receptor on the thyroid cells [3]. Uncontrolled hyperthyroidism carries significant morbidity and exerts a toll on quality of life and reproductive outcomes [4, 5, 6, 7]. About 10%–30% of patients with Graves' disease have thyroid eye disease (TED) which affects physical and psychological well‐being [8]. In addition, hyperthyroidism increases cardiovascular risk including cardiac arrhythmias, heart failure and strokes [9]. In recent decades, the diagnosis and monitoring of Graves' disease has been simplified by the use of sensitive TRAb assays, together with automated assays for measuring thyroid hormones, that is, free triiodothyronine (FT3), free thyroxine (FT4) and thyroid stimulating hormone (TSH) [3, 10, 11]. Current treatment options, namely antithyroid drugs (ATD), radioactive iodine (RAI) and thyroidectomy, have been used successfully for decades, with established efficacy and side effect profiles [10, 11]. More recently, insights have emerged on treatment related outcomes including cardiovascular disease [12], quality of life [13] and feto‐maternal health [6].

In spite of these developments, aspects of Graves' disease management remain contentious. The last Graves' disease clinical practice surveys in the United Kingdom were conducted over a decade ago and highlighted significant variability in areas such as the choice of primary therapy, RAI administration protocols and Graves' disease treatment in pregnancy [14, 15]. Since then, the UK National Institute for Health and Care Excellence (NICE) have recommended RAI as primary therapy for Graves' disease representing a change from the traditional use of ATDs as first‐line [16]. RAI is cost effective and has been shown to confer survival benefits through effective control of hyperthyroidism [17, 18]. At the same time, RAI therapy may aggravate TED [5], and in addition, concerns have been raised regarding a possible link between RAI treatment for hyperthyroidism and the risk of solid cancers [19]. In practice, ATDs remain popular, as shown in a recent global survey in which 92% of respondents preferred ATDs as primary modality for Graves' disease [20]. In addition, some centres have reported excellent long‐term control with continuous low‐dose ATDs [21], an approach that was widely adopted during the Covid‐19 lockdown when RAI services were suspended [22]. It is unclear how these changes in evidence and guidelines have impacted upon Graves' disease treatment in the UK. Therefore, we have undertaken a nationwide survey of Graves' disease treatment and compared current practice with previous surveys.

## 2 Methods

### 2.1 Survey Distribution

Between October 2022 and March 2023, endocrinologists practicing in the United Kingdom were invited to complete a 15 min online survey on the management of Graves' disease. The survey was undertaken on behalf of the British Thyroid Association (BTA) and the survey questionnaire was reviewed by executives of the BTA and the UK Society for Endocrinology (SfE) before release. After approval, the survey was circulated by e‐mail to members of the BTA, SfE and to regional endocrinology networks. Participants could access the survey via a link embedded either within the e‐mail or attached newsletter. An initial invitation was followed‐up by a single reminder approximately 3 months later.

### 2.2 Survey Domains

The survey comprised 27 questions which covered the following domains [1]: professional details and region of respondent [2], diagnosis and choice of primary therapy [3], dose, treatment duration, and monitoring of ATDs [4], approach to recurrent disease [5], administration of RAI, and [6] management of a woman preconception and during pregnancy. To allow comparisons with previous surveys the questionnaire was designed after the 2008 questionnaire by Vaidya et al. [14], using the same index case of a 42‐year‐old woman with an initial presentation of Graves' disease, a small diffuse goitre, FT4 of 45 pmol/L and no eye signs. The same index case with minor variations has been used in other previous national and international surveys. A copy of the questionnaire is available from the authors on request.

### 2.3 Statistical Analysis

Survey data was anonymously collected using a web‐based commercial survey service, SurveyMonkey (www.surveymonkey.com). Questions on treatment choices were multiple choices with single best response while questions on diagnostic preferences allowed multiple responses. Data are summarised descriptively using numbers and percentages. Frequencies were adjusted to 100%, excluding non‐responders and results are presented as percentages. Comparisons between groups were undertaken with the chi‐squared test for categorical data and Mann–Whitney‐*U* tests or Kruskal−Wallis test for continuous data depending on the number of groups. Data was stored in a password protected web account and downloaded to an excel spreadsheet and then analysed using Stata version 17·0 for Windows (Stata Corp., College Station, TXS, USA).

## 3 Results

### 3.1 Respondent Characteristics

A total of 158 respondents completed the questionnaire comprising predominantly endocrinologists (*n* = 156, 99%) and consultants (*n* = 143, 91%) (Table 1). Respondents were spread across the UK with 61% from England, 18% from Scotland, 15% from Wales and 6% from Northern Ireland (Table 1).

**Table 1:** Characteristics of survey respondents.

Number of respondents	158
Region	
Scotland	29 (18%)
North East	29 (18%)
Wales	23 (15%)
South West	19 (12%)
London	14 (9%)
East Midlands	9 (6%)
Northern Ireland	9 (6%)
South East	8 (5%)
East of England	6 (4%)
West Midlands	6 (4%)
Other regions	6 (4%)
Specialty	
Endocrinology	156 (99%)
Surgery	2 (1%)
Position	
Consultant	143 (91%)
Specialist registrar	11 (7%)
Specialist nurse	4 (3%)
Patients seen yearly	
> 30	115 (73%)
10−30	40 (25%)
< 10	3 (2%)

*Note:* Figures are numbers (% of total). Percentages may not add up to 100 due to rounding.

### 3.2 Diagnostic Evaluation of the Index Case

**Figure 1:**
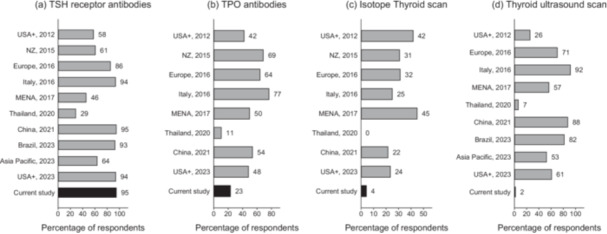
Diagnostic evaluation of index case: UK and international surveys. Figures are percentages of the total number of respondents who would request (a) TSH receptor antibodies, (b) TPO antiboies, (c) Isotope thyroid scan, and (d) Thyroid ultrasound scan. Surveys were from New Zealand (NZ), 2015 [23], Europe, 2016 [24], Italy, 2016 [25], Middle East and North Africa (MENA), 2017 [26], Thailand, 2020 [27], China, 2021 [28], Brazil, 2023 [29] and Asia Pacific, 2023 [30]. USA+, 2012 [31] and USA+, 2023 [20], are international surveys from multiple regions including North America, South America, MENA and Asia.

For the evaluation of the index case of a 42‐year‐old woman with a first presentation of Graves' disease, 150 (95%) respondents would request a TRAb test while 37 (23%) requested thyroid peroxidase antibodies (TPOAb) (Figure 1). In contrast, isotope thyroid scan and thyroid ultrasound scans were only requested by 4%, and 2%, of respondents respectively (Figure 1). The preference for TRAbs in this survey was higher than in previous international surveys (2012−2020) [23, 24, 25, 26, 27, 31] and consistent with more recent international surveys (2021−2024) [20, 28, 29, 30] (Figure 1). Compared to international surveys, requests for isotope scans and ultrasound were particularly low (< 5%) in this survey although there has been a global decline in the use of isotope scans in general [20, 23, 24, 25, 26, 27, 28, 29, 30, 31]. The preference for TRAbs did not vary according to annual number of patients seen by respondent (> 30 vs. < 30, *p* > 0.05) or by country of respondent (England, Scotland, Wales and Northern Ireland, *p* > 0.05).

### 3.3 Choice of Therapy

**Figure 2:**
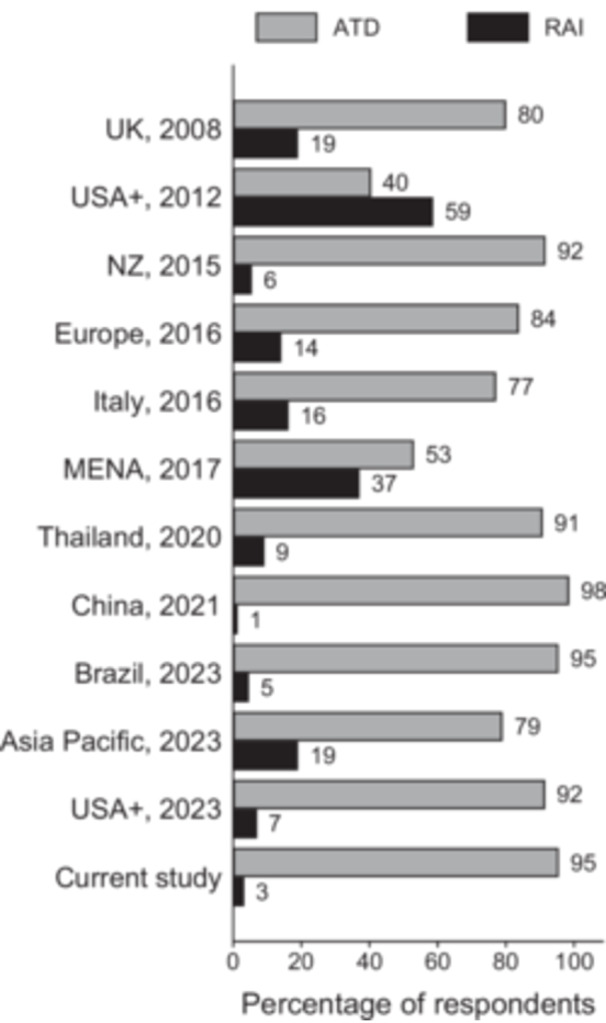
Preferred primary treatment: UK and international surveys. Figures are percentages of the total number of respondents. Surveys were from UK, 2008 [14], New Zealand (NZ), 2015 [23], Europe, 2016 [24], Italy, 2016 [25], Middle East and North Africa (MENA), 2017 [26], Thailand, 2020 [27], China, 2021 [28], Brazil, 2023 [29] and Asia Pacific, 2023 [30]. USA +, 2012 [31] and USA +, 2023 [20], are international surveys from multiple regions including North America, South America, MENA and Asia. ATD, antithyroid drug, RAI, radioactive iodine.

ATDs were the preferred first line treatment for 95% of respondents with RAI selected by only 3% and surgery by 2% of respondents (Figure 2). This represents an increase in ATD use from the previous 2008 UK survey in which 80% of respondents opted for ATD while 19% preferred RAI [14]. Preference for ATDs did not vary according to country of respondent (*p* > 0.05) or number of patients seen (*p* > 0.05). The declining trend in RAI use for primary therapy was also observed globally including the recent 2023 study in which only 7% selected RAI compared to 59% in a similar survey in 2012 [20, 31] (Figure 2). Carbimazole (CMZ) was preferred to Propylthiouracil (PTU) by all respondents who chose ATDs for primary therapy. Of this, 64% of respondents favoured using initial starting doses of 30−40 mg daily and 32% preferred 20−25 mg daily (supplementary Table 1). Majority (86%) would use a dose titration regimen while only 14% would use ATDs in conjunction with Levothyroxine, that is, the block and replace regimen. The most popular duration of ATD use was 18 months (47%), followed by 12 months (24%) while 16% of participants would continue ATDs until such a time as TRAbs became negative (Table S1). For recurrent disease, 7% of respondents would still use ATDs, 81% would opt for RAI and 8% would leave the choice of treatment to the patient. For both a 19‐year‐old and a 91‐year‐old patient, 87% of respondents preferred ATDs while 12% selected RAI.

### 3.4 ATD Monitoring

After initiating ATDs, 83% of respondents would re‐check thyroid function tests in 4−6 weeks and 17% in 2−3 months (supplementary Table 1). TRAbs would be re‐checked during follow‐up by 76% of respondents, including 47% who would re‐check TRAbs when planning to stop treatment (Table S1). For patients on CMZ or PTU, 11% of respondents would request 3−6 monthly full blood counts while 28% would only check blood counts at baseline and 53% would not check at all (Table S1). Liver enzymes for patients on CMZ would be monitored every 3−6 months by 18%, checked only at baseline by 26%, and not checked at all by 54% of respondents. For PTU, liver enzymes would be monitored every 3−6 months by 44%, at baseline only by 20%, and not at all by 30%. If patients develop a rash while on CMZ, 4% of respondents would continue CMZ, 75% would switch to PTU, while 8% would switch to an alternative treatment modality (Table S1).

### 3.5 RAI Treatment Choices

**Figure 3:**
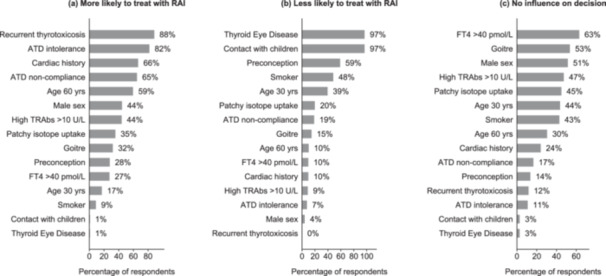
Factors influencing the decision to treat with radioactive iodine. Figures are percentages of the total number of respondents who selected various factors as being (a) more likely to lead to treatment with RAI, (b) less likely to lead to treatment with RAI, or (c) not influencing their decision to treat with RAI. ATD, antithyroid drug; RAI, radioiodine.

Respondents were asked to rate factors which would influence or not influence their decision to use RAI. The most frequently selected factors against choosing RAI were the presence of TED (97%), contact with young children (97%) or being a female intending to start a family (59%) (Figure 3). The most frequent options in favour of RAI were recurrence of thyrotoxicosis (88%), medication intolerance (82%) or non‐compliance (65%) and a cardiac history (66%) (Figure 3). Top factors selected as having no influence on the decision to use RAI were FT4 > 40 pmol/L (63%), goitre (53%), male sex (51%) and TRAb level > 10 IU/L (47%) (Figure 3). Regarding TED, 96% would avoid radioiodine in active TED while 4% would use it with steroid cover in active TED. In inactive TED, 23% would avoid RAI while 53% would use it with steroid cover and 19% would use it without steroid cover (Table S2).

### 3.6 RAI Administration

Only 3% of respondents would administer a calculated dose of RAI while 80% would administer a fixed dose typically ranging from 400−600 MBq (Table S2). Before RAI treatment, 76% of respondents would use supplementary treatment with ATDs, either prescribed alone (21%), or in combination with betablockers (51%) or Levothyroxine (4%). Before RAI administration, respondents usually stopped ATDs for durations ranging from < 7 days (41%), to 7−14 days (51%) and 15−21 days (3%) (Table S2). After RAI treatment, 61% would not use any supplementary treatment while 29% of respondents would use supplementary treatment comprising ATD alone (10%), ATD plus betablockers (5%), ATD plus Levothyroxine (11%), or Levothyroxine alone (3%) (Table S2).

### 3.7 Surgery

In preparation for surgery, 34% of respondents would routinely administer potassium iodide (KI) and beta‐blockers while 66% would not use pre‐operative medications.

### 3.8 Pregnancy and Preconception

**Figure 4:**
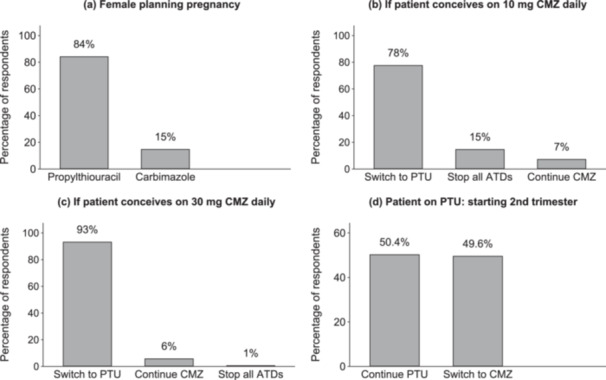
Choice of antithyroid drugs (ATD) in pregnancy. Figures are percentages of respondents who selected various ATD options for the management of a woman with Graves' disease (a) who is planning pregnancy in the next 12‐months, (b) who becomes pregnant on low dose carbimazole (CMZ), (c) who becomes pregnant on high‐dose CMZ, and (d) patient on Propylthiouracil (PTU) who is starting the second trimester. Percentages are of the total number of answered responses but may not total 100 either due to rounding or the inclusion of equivocal answers by respondents.

For a woman planning pregnancy in the next 12 months, 63% of respondents selected ATDs while thyroidectomy and RAI was preferred by 18% and 11%, respectively. PTU was the preconception ATD chosen by 84% of respondents while 15% preferred CMZ (Figure 4). Women who conceive on low‐dose CMZ (10 mg) would be switched to PTU by 78% of respondents, while 15% would stop all ATDs, and 7% would continue CMZ. For women who conceive on 30 mg of CMZ, 93% would switch to PTU, 6% would continue CMZ and 1% would stop all ATDs. Half of respondents would switch back from PTU to CMZ in the second trimester while the other half would continue PTU beyond the first trimester (Figure 4). Choice of ATD at preconception, conception, and second‐trimester stages, did not vary according to country of respondent (*p* > 0.05) or annual number of patients seen by respondent (*p* > 0.05).

## 4 Discussion

We have evaluated contemporary management of Graves' disease amongst UK endocrine professionals in light of recent fluxes and uncertainties in evidence and guidelines. The results show a growing preference for the use of TRAbs for diagnosis and prognosis in preference to isotope studies or ultrasound scans. We observed an increasing preference for first‐line treatment with ATDs rather than RAI. Nonetheless, RAI remains the treatment of choice for recurrent disease although 7% of participants would continue to use ATDs after relapse. RAI was also favoured in patients with cardiac disease and those with thionamide intolerance or non‐compliance while the presence of TED, contact with children, and preconception planning were the main factors weighing against RAI use. PTU was the predominant thionamide choice in the preconception and early gestation period, but thionamide choice beyond the first trimester remains controversial, with a 50% split between CMZ and PTU use.

Current NICE guidance advocates the use of RAI as first‐line treatment largely based on cost‐effectiveness data [16]. A single outpatient dose of RAI effectively controls hyperthyroidism in over 80%−85% of cases [32]. In the long‐term, early and effective use of RAI improves cardiovascular morbidity and mortality outcomes compared to ATDs [12]. NICE is the first major guideline to recommend primary RAI therapy, unlike other international guidelines which recommend ATDs [10] or a choice of all treatment modalities depending on patient factors [11]. Thus, the increasing preference for ATDs in the UK remains consistent with global trends. Practically every published national survey over the last 5 years report a high preference for ATDs even in areas where the use of RAI was previously the norm [20, 28, 29, 30]. The example of North America is particularly striking as RAI was the predominant modality in the region just over a decade ago [31], but presently the proportion of respondents who would now use ATDs as primary therapy has doubled, from 41% in 2012 to 86% in 2023 [20].

The reasons for discordance between guidelines and practice in the UK are unclear. Contrary to expectation, respondents' choice of primary therapy in our survey was not influenced by traditional predictors of remission such as biochemical severity, goitre, male sex or TRAb levels. Instead, more pragmatic factors like the presence of TED, pregnancy intention or contact with young children mitigated against RAI use. RAI may aggravate TED and is contraindicated in pregnancy and early life due to the risk of harm from radioisotopes. To compound matters, a highly publicised 2019 paper raised concern regarding potential cancer risk in RAI‐treated patients with hyperthyroidism [19]. While expert bodies and subsequent studies have robustly refuted this association [33, 34, 35], it is not inconceivable that RAI safety concerns have continued to influence practice. Furthermore, some patients may prefer ATDs in a bid to avoid post‐ablative hypothyroidism. Lastly, centres without immediate access to nuclear medicine facilities may opt for ATDs as a more accessible treatment modality. Indeed, ATDs were successfully used for primary and recurrent Graves' disease during the Covid‐19 pandemic buttressing the growing experience from centres on the benefits of long‐term low‐dose ATDs as a viable therapeutic strategy [21, 22].

The preference for ATDs over RAI in contradiction to the NICE recommendations will need to be explored further. Observational studies of practice trends will be required to determine whether our survey is a true reflection of practice or whether the survey respondents simply represent a section of endocrinologists biased towards ATDs. In addition, qualitative studies could provide further insights. The discordance between practice and guidelines may simply suggest a difference in emphasis between NICE with its cost‐effectiveness focus and clinicians who may tend towards a more individualised approach. A pragmatic option may be to withhold the decision to use RAI until after an initial 2−3 months of ATDs to allow the endocrinologist to establish rapport with the patient and evaluate remission risk before discussing definitive therapy. This approach would still be somewhat consistent with NICE which recommends that ATDs can be used initially to control hyperthyroidism pending primary therapy with RAI [16].

Only 2% of our survey respondents would offer thyroidectomy to the uncomplicated index case. Although surgery was not a popular treatment modality in the survey, it may have a role in patients with large goitres or co‐existent thyroid nodules where there is a risk of malignancy [11]. Thyroidectomy may also be used in instances where definitive treatment is indicated but the patient wishes to avoid RAI for example, patients with TED, individuals planning to have children in the near future, or those who are unable to comply with RAI restrictions [11]. A small number of studies have evaluated the combined use of thyroidectomy and post‐surgical RAI ablation of active thyroid remnants, as a means of improving TED, but this approach is not practised in the United Kingdom [36, 37].

Some aspects of ATD monitoring also deviated from guidelines. For example, 11% would monitor ATDs with 3−6 monthly blood counts, while liver enzymes were routinely monitored in patients on CMZ and PTU by 18% and 44% of respondents, respectively. However, the value of periodic liver and blood count monitoring in preventing thionamide side effects is debatable and routine monitoring is not supported by current NICE guidelines [16]. A new development however is the adoption of TRAbs as the key aetiological diagnostic tool, a trend which is also observed globally. It is notable that preference for isotopes and ultrasound in the UK is much lower than in other parts of the world [20, 23, 24, 25, 26, 27, 28, 29, 30, 31]. Previous UK studies have shown that isotope studies provide limited additional diagnostic information over that obtained from clinical examination and TRAb data [38]. With modern 2nd or 3rd generation assays, TRAbs exhibit sensitivity and specificity rates approaching 100%, and are also useful in predicting disease recurrence [3].

In this survey, TRAbs were also considered helpful for prognosis and would be checked at follow‐up by 76% of participants including 47% who would use TRAb status to determine when to stop ATDs. In the literature, baseline TRAb cut‐off values ranging from 5.0 to 46.5 IU/L have been reported to predict disease recurrence after initial ATD therapy [39, 40, 41, 42]. TRAbs have also been combined with other clinical parameters in predictive models. The GREAT score which combines age, goitre, FT4 and TRAb activity reports relapse rates of 74% in the high‐risk category compared to 34% in the lowest‐risk category [39]. TRAb normalisation before stopping therapy also predicts a higher chance of sustained remission with relapse rates of 80%−100% in patients with persistently elevated TRAbs compared to 20‐30% in those with negative or low TRAb activity [43, 44]. Lastly, a 2018 study from the European Group on Graves Orbitopathy (EUGOGO) showed that high TRAb activity in patients with Graves' disease predicted the development of TED [45].

We observed a varied approach to preconception and pregnancy treatment probably reflecting uncertainties in the current state of the evidence. ATDs are the mainstay of treatment in pregnancy since RAI is contraindicated and thyroidectomy is avoided in pregnancy [46]. Thus, women with Graves' disease who are planning pregnancy have a choice of continuing ATDs, or alternatively, first ablating the thyroid with RAI or surgery before embarking on pregnancy. RAI would however require a minimum wait of 6−12‐months before conception while surgery may be considered a major undertaking in the lead up to pregnancy. Furthermore, women who were treated with RAI or thyroidectomy have been shown to have a higher prevalence of thyroid dysfunction in subsequent pregnancy compared to those treated with ATD before pregnancy [47]. In our survey, 63% of respondents chose to continue preconception ATDs while 11% and 18%, respectively, opted for RAI or surgery.

PTU was the preferred preconception drug, chosen by 84% of respondents, majority of whom would also switch from CMZ to PTU on conception. The use of PTU in our survey is higher than figures reported in the recent international survey by Villagelin et al where PTU was preferred preconception by 50% of respondents [20]. These discrepancies may be due to differences in regulatory emphasis. CMZ is subject to UK Medicines and Healthcare products Regulatory Agency (MHRA) advice on the need for contraception which could lead UK clinicians to avoid its use in the preconception stage [16]. In the United States on the other hand, PTU use could be discouraged by the FDA black box warning on the risk of PTU‐induced liver dysfunction [46].

Antenatal thionamide exposure carries small risks of congenital anomalies which are more severe and more prevalent for CMZ than for PTU [48, 49]. Accordingly, guidelines recommend using PTU in early gestation to reduce fetal anomaly risk and then to consider switching back to CMZ in subsequent trimesters with the aim of reducing the odds of PTU‐induced liver dysfunction [46]. However, switching from CMZ to PTU in early gestation has not been proven to reduce anomaly risk in large cohort studies, with one meta‐analysis reporting strikingly higher anomaly rates in women who switch compared to single thionamide exposure [50]. Thus, although the majority of our respondents would switch from CMZ to PTU on conception, 15% would opt to stop all ATDs rather than switch to PTU in women who conceive on low‐dose CMZ. Also uncertain was the decision to switch back to CMZ after the first trimester which was evenly split at 50−50 amongst our respondents. Changing medications mid‐gestation may be inconvenient and may lead to deterioration of thyroid control during the switch. In the international survey by Villagelin et al, only 30% of international respondents would continue on PTU [20], further confirming the inclination towards PTU in the UK. Further studies are therefore required to clarify fetal and maternal outcomes according to different thionamides, timing of switch, disease severity and dose response.

Ours is the first comprehensive survey of Graves' disease practice in the UK for over a decade. The survey has highlighted several important trends in light of developments in research and guidelines. A limitation of our survey is its relatively small sample size. UK specialist data suggests the existence of about 1400 diabetes and endocrinology specialists in 2023, of which 75% (1050) would reasonably be expected to actively manage patients with thyroid disorders [51]. Thus, we estimate that our sample size of 158 represents about 15% of potential participants. This figure is consistent with national surveys which have reported response rates of 4%−25% of the relevant specialist groups [20]. Nonetheless, our survey respondents were distributed widely across the four UK nations and would thus be representative of present day practice. Lastly, over 70% of our respondents saw 30 patients or more each year confirming an appropriate workload in thyroid disease management.

In conclusion, the current survey highlights contemporary trends in the care of patients with Graves' disease across the UK notably the increasing diagnostic and prognostic use of TRAbs, declining use of radioisotopes and the overwhelming preference for ATDs as the primary choice of therapy. This is consistent with global treatment trends and appears to be driven more by pragmatic considerations rather than UK guideline recommendations. These findings will need to be confirmed in real world studies involving recent treatment cohorts to ensure that the survey is actually representative of practice across the country. Studies will be required to explore the decision making processes amongst clinicians and their patients to understand what factors carry the greatest value for patients in arriving at treatment choices. Lastly, the survey highlights the need for further well‐designed research in areas such as cost‐effectiveness, acceptability of existing therapies to patients, and long‐term treatment‐related outcomes including cardiovascular disease, cancer and feto‐maternal consequences of treatment.

## Conflicts of Interest

The authors declare no conflicts of interest.

## Supporting information

Table S1.

Table S2.

## References

[1] L. Chaker , D. S. Cooper , J. P. Walsh , and R. P. Peeters , “Hyperthyroidism,” Lancet 403, no. 10428 (2024): 768–780. 38278171 10.1016/S0140-6736(23)02016-0

[2] P. N. Taylor , D. Albrecht , A. Scholz , et al., “Global Epidemiology of Hyperthyroidism and Hypothyroidism,” Nature Reviews Endocrinology 14, no. 5 (2018): 301–316. 10.1038/nrendo.2018.18 29569622

[3] G. J. Kahaly , T. Diana , and P. D. Olivo , “TSH Receptor Antibodies: Relevance & Utility,” Endocrine Practice: Official Journal of the American College of Endocrinology and the American Association of Clinical Endocrinologists 26, no. 1 (2020): 97–106. 32022598 10.4158/EP-2019-0363

[4] O. M. Bello , M. Druce , and E. Ansari , “Graves' Ophthalmopathy: The Clinical and Psychosocial Outcomes of Different Medical Interventions—A Systematic Review,” BMJ Open Ophthalmology 9, no. 1 (2024): e001515. 10.1136/bmjophth-2023-001515 PMC11184183 38886120

[5] W. M. Wiersinga , K. G. Poppe , and G. Effraimidis , “Hyperthyroidism: Aetiology, Pathogenesis, Diagnosis, Management, Complications, And Prognosis,” Lancet Diabetes & Endocrinology 11, no. 4 (2023): 282–298. 36848916 10.1016/S2213-8587(23)00005-0

[6] O. E. Okosieme , I. Khan , and P. N. Taylor , “Preconception Management of Thyroid Dysfunction,” Clinical Endocrinology 89, no. 3 (2018): 269–279. 29706030 10.1111/cen.13731

[7] M. Abraham‐Nordling , O. Törring , B. Hamberger , et al., “Graves' Disease: A Long‐Term Quality‐of‐Life Follow Up Of Patients Randomized to Treatment With Antithyroid Drugs, Radioiodine, or Surgery,” Thyroid 15, no. 11 (2005): 1279–1286. 16356093 10.1089/thy.2005.15.1279

[8] H. B. Burch , P. Perros , T. Bednarczuk , et al., “Management of Thyroid Eye Disease: A Consensus Statement by the American Thyroid Association and the European Thyroid Association,” Thyroid 32, no. 12 (2022): 1439–1470. 36480280 10.1089/thy.2022.0251 PMC9807259

[9] F. Brandt , A. Green , L. Hegedüs , and T. H. Brix , “A Critical Review And Meta‐Analysis of the Association between Overt Hyperthyroidism and Mortality,” European Journal of Endocrinology 165, no. 4 (2011): 491–497. 21724839 10.1530/EJE-11-0299

[10] G. J. Kahaly , L. Bartalena , L. Hegedüs , L. Leenhardt , K. Poppe , and S. H. Pearce , “2018 European Thyroid Association Guideline for the Management of Graves' Hyperthyroidism,” European Thyroid Journal 7, no. 4 (2018): 167–186. 30283735 10.1159/000490384 PMC6140607

[11] D. S. Ross , H. B. Burch , D. S. Cooper , et al., “2016 American Thyroid Association Guidelines for Diagnosis and Management of Hyperthyroidism and Other Causes of Thyrotoxicosis,” Thyroid 26, no. 10 (2016): 1343–1421. 27521067 10.1089/thy.2016.0229

[12] O. E. Okosieme , P. N. Taylor , C. Evans , et al., “Primary Therapy Of Graves' Disease and Cardiovascular Morbidity and Mortality: A Linked‐Record Cohort Study,” Lancet Diabetes & Endocrinology 7, no. 4 (2019): 278–287. 30827829 10.1016/S2213-8587(19)30059-2

[13] A. E. Meling Stokland , M. Austdal , B. G. Nedrebø , et al., “Outcomes of Patients With Graves Disease 25 Years After Initiating Antithyroid Drug Therapy,” Journal of Clinical Endocrinology & Metabolism 109, no. 3 (2024): 827–836. 37747433 10.1210/clinem/dgad538 PMC10876387

[14] B. Vaidya , G. R. Williams , P. Abraham , and S. H. S. Pearce , “Radioiodine Treatment for Benign Thyroid Disorders: Results of a Nationwide Survey of UK Endocrinologists,” Clinical Endocrinology 68, no. 5 (2008): 814–820. 17973939 10.1111/j.1365-2265.2007.03097.x

[15] J. Hookham , E. E. Collins , A. Allahabadia , and S. P. Balasubramanian , “Variation in the Use of Definitive Treatment Options in The Management of Graves' Disease: A UK Clinician Survey,” Postgraduate Medical Journal 93, no. 1098 (2017): 198–204. 27531964 10.1136/postgradmedj-2016-134187

[16] NICE , Thyroid Disease Guidelines (National Institute of Health and Care Excellence, 2019), https://www.nice.org.uk/guidance/indevelopment/gid-ng10074/documents.

[17] O. E. Okosieme , P. N. Taylor , and C. M. Dayan , “Should Radioiodine Now be First Line Treatment for Graves' Disease?,” Thyroid Research 13 (2020): 3. 32165924 10.1186/s13044-020-00077-8 PMC7061474

[18] P. J. Donovan , D. S. A. McLeod , R. Little , and L. Gordon , “Cost‐Utility Analysis Comparing Radioactive Iodine, Anti‐Thyroid Drugs and Total Thyroidectomy for Primary Treatment of Graves' Disease,” European Journal of Endocrinology 175, no. 6 (2016): 595–603. 27634939 10.1530/EJE-16-0527

[19] C. M. Kitahara , A. Berrington de Gonzalez , A. Bouville , et al., “Association of Radioactive Iodine Treatment With Cancer Mortality in Patients With Hyperthyroidism,” JAMA Internal Medicine 179 (2019): 1034. 31260066 10.1001/jamainternmed.2019.0981 PMC6604114

[20] D. Villagelin , D. S. Cooper , and H. B. Burch , “A 2023 International Survey of Clinical Practice Patterns in the Management of Graves Disease: A Decade of Change,” Journal of Clinical Endocrinology & Metabolism 109, no. 11 (2024): 2956–2966. 38577717 10.1210/clinem/dgae222 PMC12102715

[21] M. J. Levy , N. Reddy , D. Price , et al., “Audit of Long‐Term Treatment Outcomes of Thyrotoxicosis in a Single‐Centre Virtual Clinic: The Utility of Long‐Term Antithyroid Drugs,” Clinical Endocrinology 97, no. 5 (2022): 643–653. 35274339 10.1111/cen.14721 PMC9790704

[22] K. Boelaert , W. E. Visser , P. N. Taylor , C. Moran , J. Léger , and L. Persani , “Endocrinology in the Time of Covid‐19: Management of Hyperthyroidism and Hypothyroidism,” European Journal of Endocrinology 183, no. 1 (2020): G33–G39. 32438340 10.1530/EJE-20-0445 PMC7938012

[23] S. C. Cox , J. A. Tamatea , J. V. Conaglen , and M. S. Elston , “The Management of Graves' Disease in New Zealand 2014,” New Zealand Medical Journal 129, no. 1436 (2016): 10–24. 27355225

[24] L. Bartalena , H. B. Burch , K. D. Burman , and G. J. Kahaly , “A 2013 European Survey of Clinical Practice Patterns in the Management of Graves' Disease,” Clinical Endocrinology 84, no. 1 (2016): 115–120. 25581877 10.1111/cen.12688

[25] R. Negro , R. Attanasio , F. Grimaldi , R. Guglielmi , and E. Papini , “A 2015 Italian Survey of Clinical Practice Patterns in the Management of Graves' Disease: Comparison With European and North American Surveys,” European Thyroid Journal 5, no. 2 (2016): 112–119. 27493885 10.1159/000444482 PMC4949368

[26] S. A. Beshyah , A. B. Khalil , I. H. Sherif , et al., “A Survey of Clinical Practice Patterns in Management of Graves Disease in The Middle East and North Africa,” Endocrine Practice 23, no. 3 (2017): 299–308. 27967219 10.4158/EP161607.OR

[27] C. Sriphrapradang , “Diagnosis and Management of Graves' Disease in Thailand: A Survey of Current Practice,” Journal of Thyroid Research 2020 (2020): 1–8. 10.1155/2020/8175712 PMC7238361 32454522

[28] X. Wang , X. Teng , C. Li , et al., “A Chinese Survey on Clinical Practice in Hyperthyroidism Management: Comparison With Recent Studies and Guidelines,” Endocrine Connections 10, no. 9 (2021): 1091–1100. 34382578 10.1530/EC-21-0340 PMC8494401

[29] D. Villagelin , G. M. F. S. Mazeto , C. O. Mesa , et al., “Treatment of Graves' Disease in Brazil: Results of a Survey Among Endocrinologists,” Archives of Endocrinology and Metabolism 67, no. 6 (2023): e000657. 37364155 10.20945/2359-3997000000657 PMC10661007

[30] R. Parameswaran , M. C. de Jong , J. L. W. Kit , et al., “2021 Asia‐Pacific Graves' Disease Consortium Survey of Clinical Practice Patterns in the Management of Graves’ Disease,” Endocrine 79, no. 1 (2023): 135–142. 36129592 10.1007/s12020-022-03193-7

[31] H. B. Burch , K. D. Burman , and D. S. Cooper , “A 2011 Survey of Clinical Practice Patterns in the Management of Graves' Disease,” Journal of Clinical Endocrinology & Metabolism 97, no. 12 (2012): 4549–4558. 23043191 10.1210/jc.2012-2802

[32] D. S. Ross , “Radioiodine Therapy for Hyperthyroidism,” New England Journal of Medicine 364, no. 6 (2011): 542–550. 21306240 10.1056/NEJMct1007101

[33] P. N. Taylor , O. E. Okosieme , K. Chatterjee , and K. Boelaert , Executive Committees of the Society for E, the British Thyroid A ., “Joint Statement From the Society for Endocrinology and the British Thyroid Association Regarding ‘Association of Radioactive Iodine Treatment With Cancer Mortality in Patients With Hyperthyroidism’,” Clinical Endocrinology 92, no. 3 (2020): 266–267. 31788839 10.1111/cen.14136

[34] N. Gronich , I. Lavi , G. Rennert , and W. Saliba , “Cancer Risk After Radioactive Iodine Treatment for Hyperthyroidism: A Cohort Study,” Thyroid 30, no. 2 (2020): 243–250. 31880205 10.1089/thy.2019.0205

[35] S. R. Shim , C. M. Kitahara , E. S. Cha , S. J. Kim , Y. J. Bang , and W. J. Lee , “Cancer Risk After Radioactive Iodine Treatment for Hyperthyroidism: A Systematic Review and Meta‐Analysis,” JAMA Network Open 4, no. 9 (2021): e2125072. 34533571 10.1001/jamanetworkopen.2021.25072 PMC8449277

[36] M. Oeverhaus , J. Koenen , N. Bechrakis , et al., “Radioiodine Ablation of Thyroid Remnants in Patients With Graves' Orbitopathy,” Journal of Nuclear Medicine 64, no. 4 (2023): 561–566. 36418167 10.2967/jnumed.122.264660

[37] A. De Bellis , G. Conzo , G. Cennamo , et al., “Time Course of Graves' Ophthalmopathy After Total Thyroidectomy Alone or Followed by Radioiodine Therapy: A 2‐Year Longitudinal Study,” Endocrine 41, no. 2 (2012): 320–326. 22169963 10.1007/s12020-011-9559-x

[38] O. E. Okosieme , D. Chan , S. A. Price , J. H. Lazarus , and L. D. K. E. Premawardhana , “The Utility of Radioiodine Uptake and Thyroid Scintigraphy in the Diagnosis and Management of Hyperthyroidism,” Clinical Endocrinology 72, no. 1 (2010): 122–127. 19453641 10.1111/j.1365-2265.2009.03623.x

[39] X. G. Vos , E. Endert , A. H. Zwinderman , J. G. P. Tijssen , and W. M. Wiersinga , “Predicting the Risk of Recurrence Before the Start of Antithyroid Drug Therapy in Patients With Graves’ Hyperthyroidism,” Journal of Clinical Endocrinology & Metabolism 101, no. 4 (2016): 1381–1389. 26863422 10.1210/jc.2015-3644

[40] T. Struja , M. Kaeslin , F. Boesiger , et al., “External Validation of the GREAT Score to Predict Relapse Risk in Graves' Disease: Results From a Multicenter, Retrospective Study With 741 Patients,” European Journal of Endocrinology 176, no. 4 (2017): 413–419. 28100628 10.1530/EJE-16-0986

[41] J. Karmisholt , S. L. Andersen , I. Bulow‐Pedersen , A. Carlé , A. Krejbjerg , and B. Nygaard , “Predictors of Initial and Sustained Remission in Patients Treated With Antithyroid Drugs for Graves' Hyperthyroidism: The RISG Study,” Journal of Thyroid Research 2019 (2019): 5945178. 30719273 10.1155/2019/5945178 PMC6335719

[42] C. Cappelli , E. Gandossi , M. Castellano , et al., “Prognostic Value of Thyrotropin Receptor Antibodies (TRAb) in Graves' Disease: A 120 Months Prospective Study,” Endocrine Journal 54, no. 5 (2007): 713–720. 17675761 10.1507/endocrj.k06-069

[43] P. Laurberg , G. Wallin , L. Tallstedt , M. Abraham‐Nordling , G. Lundell , and O. Tørring , “TSH‐Receptor Autoimmunity in Graves' Disease After Therapy With Anti‐Thyroid Drugs, Surgery, or Radioiodine: A 5‐Year Prospective Randomized Study,” European Journal of Endocrinology 158, no. 1 (2008): 69–75. 18166819 10.1530/EJE-07-0450

[44] C. Carella , G. Mazziotti , F. Sorvillo , et al., “Serum Thyrotropin Receptor Antibodies Concentrations in Patients With Graves' Disease Before, at the End of Methimazole Treatment, and After Drug Withdrawal: Evidence That the Activity of Thyrotropin Receptor Antibody and/or Thyroid Response Modify During the Observation Period,” Thyroid: Official Journal of the American Thyroid Association 16, no. 3 (2006): 295–302. 16571093 10.1089/thy.2006.16.295

[45] W. Wiersinga , M. Žarković , L. Bartalena , et al., “Predictive Score for the Development or Progression of Graves' Orbitopathy in Patients With Newly Diagnosed Graves' Hyperthyroidism,” European Journal of Endocrinology 178, no. 6 (2018): 635–643. 29650691 10.1530/EJE-18-0039

[46] E. K. Alexander , E. N. Pearce , G. A. Brent , et al., “2017 Guidelines of the American Thyroid Association for the Diagnosis and Management of Thyroid Disease During Pregnancy and the Postpartum,” Thyroid 27, no. 3 (2017): 315–389. 28056690 10.1089/thy.2016.0457

[47] C. Minassian , L. A. Allen , O. Okosieme , B. Vaidya , and P. Taylor , “Preconception Management of Hyperthyroidism and Thyroid Status in Subsequent Pregnancy: A Population‐Based Cohort Study,” Journal of Clinical Endocrinology and Metabolism 108, no. 11 (2023): 2886–2897. 37200150 10.1210/clinem/dgad276 PMC10584009

[48] S. L. Andersen , L. Knøsgaard , J. Olsen , P. Vestergaard , and S. Andersen , “Maternal Thyroid Function, Use of Antithyroid Drugs in Early Pregnancy, and Birth Defects,” Journal of Clinical Endocrinology & Metabolism 104, no. 12 (2019): 6040–6048. 31408173 10.1210/jc.2019-01343

[49] G. H. Seo , T. H. Kim , and J. H. Chung , “Antithyroid Drugs and Congenital Malformations: A Nationwide Korean Cohort Study,” Annals of Internal Medicine 168, no. 6 (2018): 405–413. 29357398 10.7326/M17-1398

[50] M. Agrawal , S. Lewis , L. Premawardhana , C. M. Dayan , P. N. Taylor , and O. E. Okosieme , “Antithyroid Drug Therapy in Pregnancy and Risk of Congenital Anomalies: Systematic Review and Meta‐Analysis,” Clinical Endocrinology 96, no. 6 (2022): 857–868. 34845757 10.1111/cen.14646

[51] RCP . Royal College of Physicians. Work Force Survey. 2023. https://www.rcp.ac.uk/improving-care/resources/snapshot-of-uk-consultant-physicians-2023/.

