# Supplementary material for: British Thyroid Association Survey of Graves' Disease Management in the UK
Source: Clin Endocrinol (Oxf). 2025 May 8;103(3):376–84. doi: 10.1111/cen.15266 (PMC12319287; doi:10.1111/cen.15266)
Supplement: Supplementary file 1 — Table S1. [file CEN-103-376-s001.docx]

| **Supplementary Table 1: Use and monitoring of Antithyroid Drugs** | |
| --- | --- |
| **Question** | **N (%)** |
| ***What starting dose of CMZ would you prescribe for the patient described?*** |  |
| 5-15 mg daily | 3 (2%) |
| 20-25 mg daily | 49 (32%) |
| 30-40 mg daily | 96 (64%) |
| Other options | 3 (2%) |
| ***After commencing ATDs when would you next evaluate thyroid hormones?*** |  |
| 4 weeks | 31 (20%) |
| 6 weeks | 96 (63%) |
| 2 months | 21 (14%) |
| 3 months | 4 (3%) |
| ***After commencing ATDs when would you check TRAbs?*** |  |
| 6 months | 4 (3%) |
| After 12 months | 28 (19%) |
| Never | 36 (24%) |
| When planning to stop ATDs | 71 (47%) |
| Would recheck in other situations^1^ | 12 (8%) |
| ***For how long would you continue ATDs?*** |  |
| 9 Months | 1 (1%) |
| 12 Months | 36 (24%) |
| 18 Months | 71 (47%) |
| 24 Months | 2 (1%) |
| Until TRAbs becomes negative | 24 (16%) |
| Other options | 17 (11%) |
| ***If the patient is on CMZ or PTU how often would you check blood counts?*** |  |
| At baseline only | 43 (28%) |
| 3-Monthly | 10 (7%) |
| 6-Monthly | 6 (4%) |
| Not routinely | 80 (53%) |
| Other options | 12 (8%) |
| ***If the patient is on CMZ, how often would you check liver-enzymes?*** |  |
| At baseline only | 40 (26%) |
| 3-Monthly | 13 (9%) |
| 6-Monthly | 13 (9%) |
| Not routinely | 81 (54%) |
| Other options | 4 (3%) |
| ***If the patient is on PTU, how often would you check liver-enzymes?*** |  |
| At baseline only | 30 (20%) |
| 3-Monthly | 47 (32%) |
| 6-Monthly | 18 (12%) |
| Not routinely | 44 (30%) |
| Other options | 9 (6%) |
| ***If the patient develops a rash with CMZ you would:*** |  |
| Continue CMZ | 6 (4%) |
| Switch to PTU | 113 (75%) |
| Select an alternative mode of treatment: RAI or surgery | 12 (8%) |
| Other options | 20 (13%) |
| 1. Other situations included the presence of thyroid eye disease, severe presentation, planning pregnancy, or planning RAI. CMZ, Carbimazole, PTU, Propylthiouracil, ATD, antithyroid drugs, RAI, radioactive iodine | |
