# Supplementary material for: British Thyroid Association Survey of Graves' Disease Management in the UK
Source: Clin Endocrinol (Oxf). 2025 May 8;103(3):376–84. doi: 10.1111/cen.15266 (PMC12319287; doi:10.1111/cen.15266)
Supplement: Supplementary file 2 — Table S2. [file CEN-103-376-s002.docx]

| **Supplementary Table 2: Radioactive Iodine administration** | |
| --- | --- |
| **Question** | **N (%)** |
| ***RAI dose: Which of the following best reflects your practice regarding RAI?*** |  |
| Administer a calculated dose based on factors like thyroid gland volume and RAI uptake | 5 (3%) |
| Administer a fixed dose for all patients with hyperthyroidism | 46 (32%) |
| Administer a fixed dose for most patients but occasionally use a higher dose for selected patients, e.g., patients with large goitres or previously unsuccessful RAI treatment | 44 (30%) |
| Administer separate fixed doses according to the diagnosis, i.e., Graves’ disease, toxic multinodular goitre, or solitary thyroid nodule | 27 (18%) |
| Other options | 24 (16%) |
| ***If you administer fixed RAI dose regimens, what dose do you typically use?*** |  |
| <400 MBq | 1 (1%) |
| 400-499 MBq | 40 (45%) |
| 500-599 MBq | 37 (42%) |
| >600 MBq | 11 (12%) |
| ***Considering the index patient described, would you routinely use any supplementation before RAI?*** |  |
| ATD + thyroxine (block and replace) | 6 (4%) |
| ATD alone | 30 (21%) |
| ATD ± beta-blocker | 72 (51%) |
| Beta-blocker alone | 3 (2%) |
| None | 24 (17%) |
| Other options | 6 (4%) |
| **If you use ATDs before RAI, how many days before RAI would you stop ATDs?** |  |
| <7 days | 43 (41%) |
| 7-14 days | 54 (51%) |
| 15-21 days | 3 (3%) |
| I don't stop ATDs before RAI | 1 (1%) |
| Other options | 4 (4%) |
| ***With regards to RAI and thyroid eye disease (TED), which of the following best reflects your practice?*** |  |
| I avoid RAI in active TED, but use in inactive TED with steroid cover | 72 (53%) |
| I avoid RAI in active TED, but use in inactive TED without steroid cover | 26 (19%) |
| I avoid RAI in any degree of TED | 31 (23%) |
| I use RAI in both active and inactive TED with steroid cover | 6 (4%) |
| ***Do you routinely use any supplementary treatment after RAI?*** |  |
| ATD + beta-blocker | 7 (5%) |
| ATD alone | 14 (10%) |
| ATD + thyroxine (block and replace) | 15 (11%) |
| Thyroxine alone | 4 (3%) |
| None | 84 (61%) |
| Other (please specify) | 14 (10%) |
| RAI, Radioactive Iodine, ATD, antithyroid drugs, MBq megabecquerel |  |
